# The role of journal editors in closing the gender gap

**DOI:** 10.1192/j.eurpsy.2023.64

**Published:** 2023-07-19

**Authors:** J. Marsh

**Affiliations:** The Lancet Psychiatry, London, United Kingdom

## Abstract

**Abstract:**

Journal editors can promote gender equality in various ways. The main ones are consideration of gender when inviting editorial board members, peer reviewers, and authors of solicited opinion pieces or reviews. As in many areas, the problem can be that the pool of suitable women is perceived to be small and the same women are asked to perform multiple tasks in addition to their academic and clinical duties. Journal editors need to seek women who are less well known but competent for the task required. To increase the pool of qualified candidates, editors should promote training using existing resources, such as online peer reviewer courses, or develop in-house initiatives, such as *The Lancet Psychiatry’s* Editorial Board Development Programme. It is important to make public commitments to gender balance, for example as part of a diversity pledge, with specific targets, and to collect and report data with regular updates. Gender balance should be an integral part of information templates in manuscript handling systems, such as asking authors and peer reviewers to consider women when recommending alternative peer reviewers. Where relevant, journal editors can also consider gender balance in their use of images, cover art, podcast or interview subjects, profiles, news and features, and social media content.

**Disclosure of Interest:**

None Declared

